# Ghrelin Alleviates Aging‐Related Cognitive Impairment by Regulating Autophagy‐Related Signaling via the SHBG/JNK1/Beclin1 Axis in Microglia

**DOI:** 10.1111/acel.70732

**Published:** 2026-09-29

**Authors:** Lixing Zhou, Ying Cheng, Wanyu Zhao, Xuelian Sun, Fengjuan Hu, Meiling Ge, Yan Zhang, Gongchang Zhang, Birong Dong, Xiaolei Liu

**Affiliations:** ^1^ The Center of Gerontology and Geriatrics/National Clinical Research Center for Geriatrics West China Hospital, Sichuan University Chengdu China

**Keywords:** autophagy, cognitive impairment, ghrelin, immunosenescence

## Abstract

Cognitive impairment (CI) associated with aging and immunosenescence is linked to metabolic alterations. Microglial senescence and dysregulated autophagy have been implicated as major contributors to this process, but the underlying metabolic regulators remain unclear. This study aimed to identify key metabolic regulators of CI and elucidate their underlying mechanisms. Human cohort analyses, older and D‐galactose (D‐gal)‐induced mouse models, and microglial cell models were integrated to investigate the metabolic regulation of CI. Clinical analyses identified ghrelin as the factor most strongly associated with CI. Older and D‐gal mice exhibited CI, microglial senescence, enhanced inflammation, and elevated ghrelin. Ghrelin treatment improved cognitive function, attenuated microglial senescence, and suppressed inflammation, whereas high‐dose ghrelin induced growth hormone secretagogue receptor (GHSR) desensitization. Sex hormone‐binding globulin (SHBG) showed mainly cytoplasmic localization, was identified as a potential mediator of ghrelin signaling in microglia, and its expression was downregulated following ghrelin treatment. Transcriptomic profiling revealed significant enrichment of autophagy‐related pathways in ghrelin‐treated microglia. Ghrelin altered autophagy‐related markers, as indicated by increased Beclin1 and LC3 and decreased p62. SHBG overexpression or JNK1 inhibition attenuated the ghrelin‐induced alterations in autophagy‐related markers and anti‐senescent effects, whereas Beclin1 overexpression partially rescued these inhibitory effects caused by SHBG overexpression. In conclusion, ghrelin alleviated aging‐related CI, with mechanistic evidence indicating that the modulation of microglial autophagy‐related mechanisms through the SHBG/JNK1/Beclin1 axis may contribute to its protective effects, thereby providing a potential therapeutic strategy for aging‐related CI.

## Graphical abstract

This study found that aging‐related cognitive impairment is accompanied by hippocampal microglial senescence. Ghrelin suppresses microglial senescence by promoting autophagy through the SHBG/JNK1/Beclin1 axis, thereby alleviating aging‐related cognitive impairment. The graphical abstract was drawn using the Figdraw.

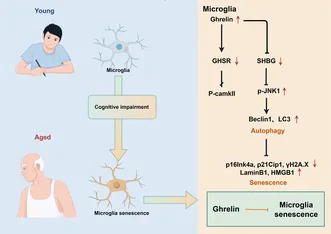

## 1 Introduction

Cognitive impairment (CI) is a prevalent, age‐related condition characterized by progressive deficits in memory, attention, and executive functions that impair activities of daily living (Gordon and Martin 2013; Tangalos and Petersen 2018). CI is clinically stratified from mild cognitive impairment (MCI), which represents an intermediate state between normal aging and dementia, to more severe forms associated with Alzheimer's disease and vascular dementia (Gordon and Martin 2013). Patients often exhibit mild but progressive impairments and are at risk of loss of independence and eventual institutionalization (Tangalos and Petersen 2018). China has the world's largest population of older adults with CI, making it a major public health challenge (Chen et al. 2024). In recent decades, large‐scale epidemiological surveys have revealed that the prevalence of CI among older adults has fluctuated over time (Chen et al. 2024). Furthermore, women and the oldest‐old are more susceptible to CI (H. Chen et al. 2024). Importantly, metabolic disturbances have increasingly been recognized as contributors to CI. Previous studies have demonstrated that metabolic syndrome (MetS) and its individual components are strongly associated with CI (Chen et al. 2019; Wang et al. 2021). As a hallmark of aging, metabolic dysregulation may contribute to cognitive decline through multiple biological pathways.

Metabolites serve as downstream indicators of diverse molecular regulatory mechanisms, reflecting age‐related phenotypic changes (Ren et al. 2024). Recent multimodal omics research indicates that metabolic remodeling is a core hallmark of aging, closely linked to immunosenescence and neurodegeneration (Ji et al. 2024). Given the associated risks, early detection through effective screening is of great significance. Previous studies have demonstrated that the Montreal Cognitive Assessment (MoCA) is more sensitive than the Mini‐Mental State Examination (MMSE) in screening for CI (Islam et al. 2023; Jia et al. 2021). Nevertheless, despite advances in screening strategies, disease‐modifying therapies for CI remain unavailable. Therefore, elucidating the mechanisms underlying metabolic dysregulation and identifying novel therapeutic targets are of critical importance.

Immunosenescence refers to the progressive decline in immune function associated with aging (Rawji et al. 2016). It is characterized by reduced immune responses to novel antigens and the accumulation of antigen‐experienced memory B and T cells (Frasca et al. 2020). In addition, a hallmark of aging in the immune system is chronic low‐grade inflammation, which is often termed “inflammaging” and results in a proinflammatory environment (Fetarayani et al. 2025). Collectively, these age‐related immune alterations impair both humoral and cellular immune responses to newly encountered antigens (Carr et al. 2025; Frasca et al. 2020). Within the central nervous system, microglia are particularly vulnerable to age‐related changes in the immune microenvironment (Prinz et al. 2019). Aged microglia exhibit a pro‐inflammatory phenotype accompanied by impaired phagocytic capacity, leading to excessive production of inflammatory cytokines, including IL‐1β and IL‐6, which exacerbate neuronal injury (Prinz et al. 2019; Sun et al. 2024). Emerging evidence further indicates that impaired autophagy promotes the senescence‐associated secretory phenotype (SASP) of microglia and accelerates amyloid pathology (Choi et al. 2023). In contrast, intact autophagic activity is essential for maintaining microglial homeostasis and preserving neuroprotective functions (Amin et al. 2023). However, it is unknown how defective autophagy promotes a senescence‐associated secretory phenotype of microglia and subsequent CI.

Ghrelin is a gut‐derived peptide hormone that exhibits neuroprotective effects in cognitive disorders. Previous studies have shown that ghrelin suppresses the release of pro‐inflammatory cytokines and ameliorates cognitive deficits in inflammatory and septic models (Wei et al. 2015). Clinical studies have reported elevated circulating ghrelin levels in patients with MCI, suggesting that ghrelin may serve as a potential biomarker of early cognitive decline (Cao et al. 2018). Mechanistically, ghrelin exerts its neuroprotective effects by attenuating oxidative stress, preserving mitochondrial function, and enhancing synaptic plasticity, thereby mitigating neurodegeneration (Yanagi et al. 2018). More recently, ghrelin was shown to inhibit ferroptosis and attenuate amyloid‐β‐induced neuroinflammation and cognitive impairment by regulating microglial polarization in Alzheimer's disease models (Guo et al. 2025). In addition, ghrelin has been reported to regulate autophagy through the AMPK/ULK1/Beclin1 signaling pathway, thereby maintaining cellular homeostasis under stress conditions (Ezquerro et al. 2017). However, whether ghrelin modulates microglial immunosenescence through autophagy and thereby alleviates aging‐related cognitive impairment remains poorly understood. In this study, we investigated the effects of ghrelin on aging‐related CI and its underlying mechanisms at the behavioral, molecular, and transcriptional levels. Our results demonstrated that ghrelin administration markedly improved learning and memory in D‐gal‐treated and older mice, while attenuating the expression of senescence markers and pro‐inflammatory cytokines in the hippocampus. Mechanistically, ghrelin restored microglial homeostasis by regulating autophagy through the sex hormone‐binding globulin (SHBG)/c‐Jun N‐terminal kinase 1 (JNK1)/Beclin1 signaling axis, thereby suppressing the senescence‐associated secretory phenotype and attenuating neuroinflammation. These findings revealed a previously unrecognized regulatory mechanism by which ghrelin modulates microglial senescence, and provided new insights into the crosstalk among metabolic signaling, immune senescence, and neurodegeneration.

## 2 Methods

### 2.1 Human Subjects

Community‐dwelling Han Chinese adults aged ≥ 50 years who underwent comprehensive cognitive assessment were recruited for this study. Exclusion criteria included (1) severe underlying medical conditions with an estimated life expectancy of < 6 months; (2) inability to complete the questionnaires or physical examinations; and (3) refusal or inability to provide written informed consent. A total of 257 participants with complete metabolomic data were included in the final analysis.

Cognitive status was classified according to Montreal Cognitive Assessment (MoCA) scores as follows: normal cognition (26–30), mild cognitive impairment (MCI; 18–25), moderate cognitive impairment (Mod‐CI; 11–17), and severe cognitive impairment (Sev‐CI; < 11) (Kihumuro et al. 2023).

This study was approved by the Biomedical Research Ethics Committee of West China Hospital, Sichuan University (Approval No. 2020‐287). Written informed consent was obtained from all participants before enrollment. The study was conducted in accordance with the Declaration of Helsinki and complied with relevant aspects of the ARRIVE guidelines.

### 2.2 Experiment 4: Additional Validation in Older Mice With Ghrelin Intervention

Mice were divided into three groups: the Young group (2‐month‐old mice), the Old group (9‐month‐old or ≥ 9‐month‐old mice), and the Old + Ghrelin group (*n* = 5 per group). Mice in the Old + Ghrelin group received intraperitoneal injections of ghrelin (80 μg/kg) once daily for four consecutive weeks, while mice in the Young and Old groups received equal volumes of 0.9% NaCl (Guo et al. 2025; Moon et al. 2011). Following the intervention period, behavioral testing was performed. Mice were then euthanized, and brain tissues were collected for subsequent analyses.

### 2.3 Mouse Model

Male C57BL/6 mice were purchased from a certified vendor and housed under standard laboratory conditions with ad libitum access to food and water. All animal experiments were approved by the Animal Ethics Committee of West China Hospital, Sichuan University (Approval No. 20240221006).

Experiment 1: Early aging model. Mice were divided into two groups: the Young group (2‐month‐old mice) and the Aged group (9‐month‐old mice) (*n* = 10 per group). Following behavioral testing, mice were euthanized, and hippocampal tissues and blood samples were collected.

Experiment 2: D‐galactose (D‐gal)‐induced aging model. Mice were randomly assigned to the Control group (0.9% NaCl) or the D‐gal group (*n* = 10 per group). In the D‐gal group, aging was induced by daily intraperitoneal injection of D‐galactose (D810319, Macklin, China; 1000 mg/kg) for eight consecutive weeks, as previously described (Hong et al. 2023; Li, Cao, et al. 2023). Control mice received an equal volume of 0.9% NaCl for the same duration. After behavioral testing, mice were euthanized, and hippocampal tissues and blood samples were collected.

Experiment 3: D‐gal‐induced aging model with ghrelin intervention. D‐gal‐treated mice were randomly assigned to four groups: D‐gal, D‐gal + Ghrelin‐Low (20 μg/kg), D‐gal + Ghrelin‐High (80 μg/kg), and Control (0.9% NaCl) (Guo et al. 2025; Moon et al. 2011) (*n* = 10 per group). Ghrelin (G274880, Aladdin, China) treatment was initiated after 4 weeks of D‐gal administration and continued once daily for an additional 4 weeks concomitantly with D‐gal treatment. Body weight was recorded weekly. Behavioral testing was performed 1 day after the final treatment, after which mice were euthanized, and hippocampal tissues were collected for subsequent analyses.

### 2.4 Novel Object Location (NOL) Test

On Day 1, mice were allowed to freely explore an empty testing chamber for 10 min to habituate to the environment. On Day 2 (familiarization phase), two identical objects were placed diagonally in the chamber, and mice were allowed to explore them for 10 min. On Day 3 (test phase), one of the two objects was relocated to a novel position while the other remained in its original location. Mice were again allowed to explore the chamber for 10 min. All trials were recorded using a digital video camera, and the exploration time for each object was quantified. Novel location preference was calculated as the ratio of the time spent exploring the object in the novel location to the total exploration time for both objects (Li, Xu, et al. 2023).

### 2.5 Novel Object Recognition (NOR) Test

On Day 1, each mouse was allowed to freely explore an empty testing chamber for 10 min to habituate to the environment. On Day 2, two identical objects (A) were placed in parallel, and mice were allowed to explore them for 10 min. On Day 3, a different pair of identical objects (C) was placed in the same positions, and mice were again allowed to explore for 10 min. On Day 4 (test phase), one of the familiar objects (C) was replaced with a novel object (D), while the other familiar object remained in the original location. Mice were allowed to explore the objects for 10 min. The exploration time for the familiar and novel objects was recorded, and novel object preference was calculated as the ratio of the time spent exploring the novel object to the total exploration time for both objects (Li, Xu, et al. 2023).

### 2.6 Morris Water Maze (MWM) Test

The Morris water maze (MWM) test was performed in a circular pool surrounded by distal visual cues. Throughout the experiment, the water temperature was maintained at 25°C, and the escape platform was submerged 1.5 cm below the water surface. During the acquisition (training) phase, each mouse was given up to 60 s to locate the hidden platform. If a mouse failed to locate the platform within the allotted time, it was gently guided to the platform and allowed to remain there for 10 s. Each mouse underwent three training trials per day for six consecutive days, with all trials conducted at the same time each day. 24 h after the final training session, a probe trial was conducted in which the platform was removed, and each mouse was allowed to swim freely for 60 s. All trials were recorded using a digital video camera, and swimming trajectories and behavioral parameters were analyzed using the SMART video‐tracking system (He et al. 2024).

### 2.7 Western Blot

Total proteins were extracted from hippocampal tissues, primary microglia, and BV2 microglial cells using RIPA lysis buffer (P0013B, Beyotime, China). Protein concentrations were quantified with the BCA assay kit (AWB0104, Abiowell, China). Equal amounts of protein were separated by SDS‐polyacrylamide gel electrophoresis (SDS‐PAGE) and transferred onto nitrocellulose membranes. After blocking with 5% skim milk in PBST, the membranes were incubated overnight at 4°C with the indicated primary antibodies. Following three washes with PBST, the membranes were incubated with horseradish peroxidase (HRP)‐conjugated secondary antibodies. Immunoreactive bands were detected using an enhanced chemiluminescence (ECL) detection kit (K‐12045‐D50, Advansta, USA) and visualized with a chemiluminescence imaging system (ChemiScope 6100, Clinx Science, China). Details of all antibodies are provided in Table 1.

**TABLE 1:** The information on the antibody.

Name	Dilution rate	Cat. number	Source	Company	Country
p16	1:1000	28416‐1‐AP	Rabbit	Proteintech	USA
p21	1:2000	10355‐1‐AP	Rabbit	Proteintech	USA
γH2AX	1:8000	ab81299	Rabbit	Abcam	UK
HMGB1	1:1000	ab18256	Rabbit	Abcam	UK
LaminB	1:20000	12987‐1‐AP	Rabbit	Proteintech	USA
GHSR	1:1000	30602‐1‐AP	Rabbit	Proteintech	USA
p‐CamKII	1:2000	ab171095	Mouse	Abcam	UK
CamKII	1:5000	ab126789	Rabbit	Abcam	UK
SHBG	1:1000	AWA42539	Rabbit	Abiowell	China
SRC	1:20000	60315‐1‐Ig	Mouse	Proteintech	USA
C5AR1	1:2000	21316‐1‐AP	Rabbit	Proteintech	USA
KLF4	1:5000	11880‐1‐AP	Rabbit	Proteintech	USA
BRCA1	1:20000	83390‐6‐RR	Rabbit	Proteintech	USA
p62	1:10000	66184‐1‐Ig	Mouse	Proteintech	USA
Beclin1	1:1000	AWA12678	Rabbit	Abiowell	China
LC3	1:3000	14600‐1‐AP	Rabbit	Proteintech	USA
p‐Raf1	1:1000	AWA10531	Rabbit	Abiowell	China
Raf1	1:2000	26863‐1‐AP	Rabbit	Proteintech	USA
p‐AKT	1:500	ab38449	Rabbit	Abcam	UK
AKT	1:10000	10176‐2‐AP	Rabbit	Proteintech	USA
p‐JNK	1:2000	80024‐1‐RR	Rabbit	Proteintech	USA
JNK	1:20000	66210‐1‐Ig	Mouse	Proteintech	USA
β‐Actin	1:5000	66009‐1‐Ig	Mouse	Proteintech	USA
ATP1A1	1:10000	14418‐1‐AP	Rabbit	Proteintech	USA
HRP goat anti‐mouse IgG	1:5000	SA00001‐1	/	Proteintech	USA
HRP goat anti‐rabbit IgG	1:6000	SA00001‐2	/	Proteintech	USA

### 2.8 Immunofluorescence Staining

Paraffin‐embedded hippocampal sections were deparaffinized, rehydrated, and subjected to heat‐induced antigen retrieval in 0.01 M sodium citrate buffer (pH 6.0). Following autofluorescence quenching and blocking with 5% bovine serum albumin (BSA) for 1 h at 37°C, sections were incubated overnight at 4°C with the first primary antibody: Iba‐1 (1:200; AWA11292, Abiowell), NeuN (1:800; AWA10318, Abiowell), or GFAP (1:800; AWA10155, Abiowell). After incubation with an HRP‐conjugated secondary antibody for 30 min at 37°C, signal amplification was performed using TSA‐520 reagent (AWI0688, Abiowell) for 10 min at room temperature in the dark.

Following antibody stripping and re‐blocking, sections were incubated overnight at 4°C with a second primary antibody: γH2AX (1:400; ab81299, Abcam), high‐mobility group box 1 (HMGB1) (1:400; AWA10032, Abiowell), p21^Cip1^ (1:200; ab188224, Abcam), p16^Ink4a^ (1:200; bs‐23797R, Bioss, China), or Lamin B1 (1:400; ab8982, Abcam). Sections were then incubated with an HRP‐conjugated secondary antibody for 30 min at 37°C, followed by fluorescence labeling using TSA‐620 reagent (AWI0690, Abiowell) for 10 min at room temperature. When triple immunofluorescence staining was performed, TSA‐570 reagent (AWI0689, Abiowell) was used for fluorescence labeling.

After a second round of antibody stripping and re‐blocking, sections were incubated overnight at 4°C with the third primary antibody: p62 (1:200; bs‐2951R, Bioss), followed by HRP‐conjugated secondary antibody for 30 min at 37°C. Signal amplification was performed using TSA‐690 reagent (AWI0691, Abiowell) for 10 min at room temperature in the dark. Nuclei were counterstained with DAPI, and slides were mounted with Fluoromount‐G. Images were captured using a fluorescence microscope (BA410E, Motic, China).

For SHBG localization, BV2 cells were fixed with 4% paraformaldehyde, permeabilized with 0.3% Triton X‐100, and blocked with 5% BSA for 1 h at room temperature. Cells were incubated overnight at 4°C with an anti‐SHBG antibody (1:50; AWA42539, Abiowell), followed by Goat anti‐Rabbit IgG Alexa Fluor 488‐conjugated secondary antibody for 1 h at room temperature. Nuclei were counterstained with DAPI, and fluorescent images were captured using a fluorescence microscope.

### 2.9 Enzyme‐Linked Immunosorbent Assay (ELISA)

Hippocampal tissues were homogenized, and plasma samples were obtained by centrifugation at 1000×*g* for 10 min at 4°C. The concentrations of IL‐1α, IL‐1β, G‐CSF, IL‐4, IL‐6, IL‐8, CXCL10, LIF, MCP1, and M‐CSF in hippocampal homogenates, and total and active (acylated) ghrelin in plasma were measured using the corresponding commercial ELISA kits, including IL‐4 (CSB‐E04634m), M‐CSF (CSB‐E04659m), and CXCL10 (CSB‐E08183m‐IS) from CUSABIO; IL‐1β (KE10003), IL‐6 (KE10007), MCP1 (KE10123), G‐CSF (KE10025), and LIF (KE10087) from Proteintech; IL‐1α (JL13044) from JONLNBIO; IL‐8 (EM1592) from FineTest; and Ghrelin (Active) (EZRGRA‐90 K) and Ghrelin (Total) (EZRGRT‐91 K) from Sigma. All assays were performed according to the manufacturer's instructions. Absorbance was measured at 450 nm using a microplate reader, and analyte concentrations were determined from standard curves generated in parallel with the samples.

### 2.10 Cell Culture and Plasmid Transfection

Primary microglial cell isolation and culture: mice were euthanized, and brains were rapidly removed and submerged in cold PBS. The meninges and blood vessels were dissected out, and the hippocampus and cortex were removed and minced. The tissues were digested with trypsin at 37°C for 30 min and passed through a 70‐μm cell strainer (N258367, Nest, China). The resulting cell suspension was centrifuged at 1000 rpm for 5 min, and the cell pellet was resuspended in PBS and seeded into culture flasks. After 1 h of incubation at 37°C, non‐adherent cells were removed, and adherent microglia were maintained in culture for 7–10 days. Microglia were subsequently harvested by shaking the culture flasks at 200 rpm for 2 h, followed by centrifugation and reseeding into new culture dishes.

BV2 cell culture: BV2 murine microglial cells (AW‐CNM081, Abiowell) were cultured in MEM medium (AW‐MC003, Abiowell) supplemented with 10% fetal bovine serum (FBS) and 1% penicillin–streptomycin (P/S). All cells were maintained at 37°C in a humidified incubator with 95% air and 5% CO_2_.

Plasmid transfection: Primary microglia and BV2 cells were transfected with control plasmids (sh‐NC and oe‐NC), knockdown plasmids (sh‐GHSR and sh‐SHBG), or overexpression plasmids (oe‐SHBG and oe‐Beclin1) using Lipofectamine 2000 (11668019, Invitrogen, USA) according to the manufacturer's instructions. All plasmids were obtained from Honorgene (China), and their sequences are listed in Table 2. After 24 h, cells were treated with 250 mM D‐gal for 24 h. For JNK inhibition, cells were pretreated with 50 nM JNK inhibitor VIII (HY‐107598, MCE, USA) for 1 h before D‐gal treatment.

**TABLE 2:** Plasmids information.

Name	Sequences (5′‐3′)	Cat. number
sh‐NC	TTCTCCGAACGTGTCACGT	HG‐shNC
sh‐GHSR	GCAGATCAGTCAGTACTGCAA	HG‐MO177330‐sh
sh‐SHBG#1	GGCTGTATTCGGCGAGATA	HG‐MO011367‐sh
sh‐SHBG#2	GCTTCTGCTGTTGCTACTA	
oe‐SHBG	N/A	HG‐MO011367
oe‐Beclin1	N/A	HG‐MO019584

Ghrelin treatment: An in vitro senescence model was established by treating primary microglia with 250 mM D‐gal for 24 h. To determine the optimal ghrelin intervention time, cells were treated with ghrelin (1 μM) for 30 min, 3 h, or 18 h during D‐gal exposure. To determine the optimal ghrelin concentration, cells were treated with 0.1, 1, or 10 μM ghrelin for 30 min during D‐gal exposure. Untreated control cells received neither D‐gal nor ghrelin.

### 2.11 Bioinformatics Analysis

Network pharmacology analysis was performed to identify potential molecular targets of ghrelin in CI. The SMILES ID of ghrelin was obtained from PubChem and submitted to the Similarity Ensemble Approach (SEA) database (https://sea.bkslab.org/) for target prediction. Additional ghrelin‐related targets were retrieved from the CTD (https://ctdbase.org/) and GeneCards (https://www.genecards.org) databases. GeneCards‐derived targets with relevance scores above the median were retained, and all targets were corrected and deduplicated using UniProt (https://www.uniprot.org). CI‐related genes were retrieved from GeneCards, NCBI (https://www.ncbi.nlm.nih.gov/), and OMIM (https://www.omim.org) using “cognitive impairment” as the search term, with GeneCards‐derived genes similarly filtered according to the median relevance score. Overlapping ghrelin‐ and CI‐related targets were identified and visualized using Venny 2.1 (https://bioinfogp.cnb.csic.es/tools/venny/). The overlapping targets were submitted to STRING (https://string‐db.org) with the organism restricted to *Homo sapiens* and an interaction score > 0.4 to construct the protein–protein interaction (PPI) network. The network was imported into Cytoscape 3.8.0 for visualization and topological analysis using NetworkAnalyzer, and highly interconnected modules were identified using the MCODE plugin. Kyoto Encyclopedia of Genes and Genomes (KEGG) and Gene Ontology (GO) enrichment analyses were performed based on the ClusterProfiler package in R. Molecular docking was carried out using ClusPro software (https://cluspro.org/) to analyze the interaction between ghrelin and SHBG. Docking models were visualized using PyMOL and LigPlot+.

For RNA sequencing (RNA‐seq), primary microglial cells were isolated and treated under experimental conditions. Total RNA was extracted, and messenger RNA (mRNA) was enriched for library construction and sequencing. Differentially expressed genes (DEGs) were identified using standard bioinformatics pipelines. The volcano plot and principal component analysis (PCA) were used for data visualization. Gene Set Enrichment Analysis (GSEA) was performed to identify significantly enriched biological processes and signaling pathways.

### 2.12 Co‐Immunoprecipitation (Co‐IP)

Cells were washed with ice‐cold phosphate‐buffered saline (PBS) and lysed in 300 μL IP lysis buffer. Cell lysates were sonicated for 1.5 min, incubated on ice for 30 min, and centrifuged at 12,000 rpm for 15 min at 4°C. The supernatants were collected for immunoprecipitation. For antibody incubation, 100 μL of cell lysate was incubated with rabbit anti‐SHBG antibody (AWA42539, Abiowell) or normal rabbit IgG (B900610, Proteintech) overnight at 4°C with gentle rotation. Protein A/G agarose beads were prewashed with IP lysis buffer and subsequently added to the antibody–lysate mixtures. After incubation for 2 h at 4°C, the beads were collected by centrifugation at 3000 rpm for 3 min. The pellet was washed four times with IP lysis buffer, and the final pellet was resuspended in 30 μL IP lysis buffer for Western blot analysis. Western blotting was performed to detect SHBG and ghrelin (GHRL; 13309‐1‐AP, Proteintech).

### 2.13 Real‐Time Quantitative PCR (RT‐qPCR)

Total RNA was extracted from mouse hippocampal tissues using TRIzol reagent (15,596,026, Thermo, USA), following the manufacturer's protocol. Complementary DNA (cDNA) was synthesized from 1 μg of total RNA using the mRNA Reverse Transcription Kit (CW2569, CWBIO, China). RT‐qPCR was performed on a Real‐Time PCR System (QuantStudio 1, ABI, USA). Each reaction mixture contained cDNA template, gene‐specific primers, 2 × SYBR Green PCR Master Mix (CW2601, CWBIO), and nuclease‐free water. β‐actin was used as an endogenous control for normalization. Primer sequences used in this study are listed in Table 3.

**TABLE 3:** Primer sequences.

Gene	Sequence	Length
M‐β‐Actin	F ACATCCGTAAAGACCTCTATGCC R TACTCCTGCTTGCTGATCCAC	223 bp
M‐AKT1	F CGCCTGCCCTTCTACAACCAG R GCATGATCTCCTTGGCATCCTC	175 bp
M‐CTSL	F ATACAGAGCCGAGTTCGCTG R CTGGAGAGACGGATGGCTTG	136 bp
M‐ERK1	F AGTCTCTGCCCTCGAAAACCA R TCCTCTACTGTGATGCGCTTG	124 bp
M‐LKB1	F TCACAGTGCCTGGTGTCGAG R AAAGCCCCAAACCCCTCCTC	127 bp
M‐MTOR	F CCTAGAAGACAGCGGGGAAG R AAGCATCTTGCCCTGAGGTT	119 bp
M‐Raf1	F GTCGTGGAGACGAGTGTCG R TCTAACTCCCGCCATCCAAG	104 bp
M‐JNK1	F GCAGTAGTCCCCACCAATACAGA R TGTCTTTTCTACCTCCACGTCT	119 bp
M‐ERK2	F GGTTGTTCCCAAATGCTGACT R CAACTTCAATCCTCTTGTGAGGG	84 bp
M‐PRKAA1	F CGGGGTCATTCTCTATGCTT R TTTAAACCACTCGTGTTCCCT	208 bp
M‐CTSD	F GCTTCCGGTCTTTGACAACCT R CACCAAGCATTAGTTCTCCTCC	113 bp
M‐RPS6KB1	F CACAGGGAAGCTGAGGACAT R CCCCCTCCTCCAGCTC	91 bp

### 2.14 Statistical Analysis

All data are presented as the mean ± standard deviation (SD) from at least three independent experiments. Statistical analysis was performed by GraphPad Prism 9.0 software. Comparisons between two groups were performed using an unpaired, two‐tailed Student's *t*‐test. Comparisons among multiple groups were analyzed using one‐way analysis of variance (ANOVA) followed by Tukey's post hoc test. For experiments involving two independent variables, two‐way ANOVA followed by Bonferroni's multiple‐comparisons test was performed. Pearson's correlation analysis was used to analyze the correlation between two variables. A *p* < 0.05 was considered statistically significant.

## 3 Results

### 3.1 Ghrelin Dysregulation Is Associated With CI in Human Subjects

Baseline characteristics of study participants are shown in Table 4. We explored the association between energy metabolism and cognitive impairment by analyzing the metabolic indicators in participants with different cognitive statuses (normal, MCI, Mod‐CI, and Sev‐CI). As shown in Figure 1A, the expression profiles of these metabolic indicators were significantly different among the four groups. Correlation analysis revealed that blood glucose, creatinine, ghrelin, and Klotho levels were significantly negatively correlated with MoCA scores, whereas basal metabolic rate showed a negative but non‐significant correlation (*p* > 0.05) (Figure 1B). In contrast, the albumin/globulin ratio and serum magnesium levels were significantly positively correlated with MoCA scores (Figure 1B). Among all metabolic indicators examined, ghrelin exhibited the strongest negative correlation with MoCA scores (*p* = 0.0004), indicating that higher circulating ghrelin levels were associated with poorer cognitive performance and greater CI severity (Figure 1B). Based on these findings, ghrelin was selected for subsequent mechanistic investigations.

**TABLE 4:** Baseline characteristics of study participants.

Variables		Normal	MCI	Mod‐CI	Sev‐CI	*p*
Sex	Male	14 (16.9%)	35 (23.3%)	5 (25.0%)	2 (50.0%)	0.3369
	Female	69 (83.1%)	115 (76.7%)	15 (75.0%)	2 (50.0%)	
Age (Year)		54.98 ± 10.38	60.79 ± 7.04	63.4 ± 8.93	57.00 ± 14.09	< 0.0001
Education	≦ 12 years	40 (48.2%)	108 (72.0%)	20 (100.0%)	4 (100.0%)	< 0.0001
	> 12 years	43 (51.8%)	42 (28.0%)	—	—	
Degree of education		4.37 ± 1.08	3.60 ± 1.03	2.30 ± 0.73	2.00 ± 0.82	< 0.0001

**FIGURE 1:**
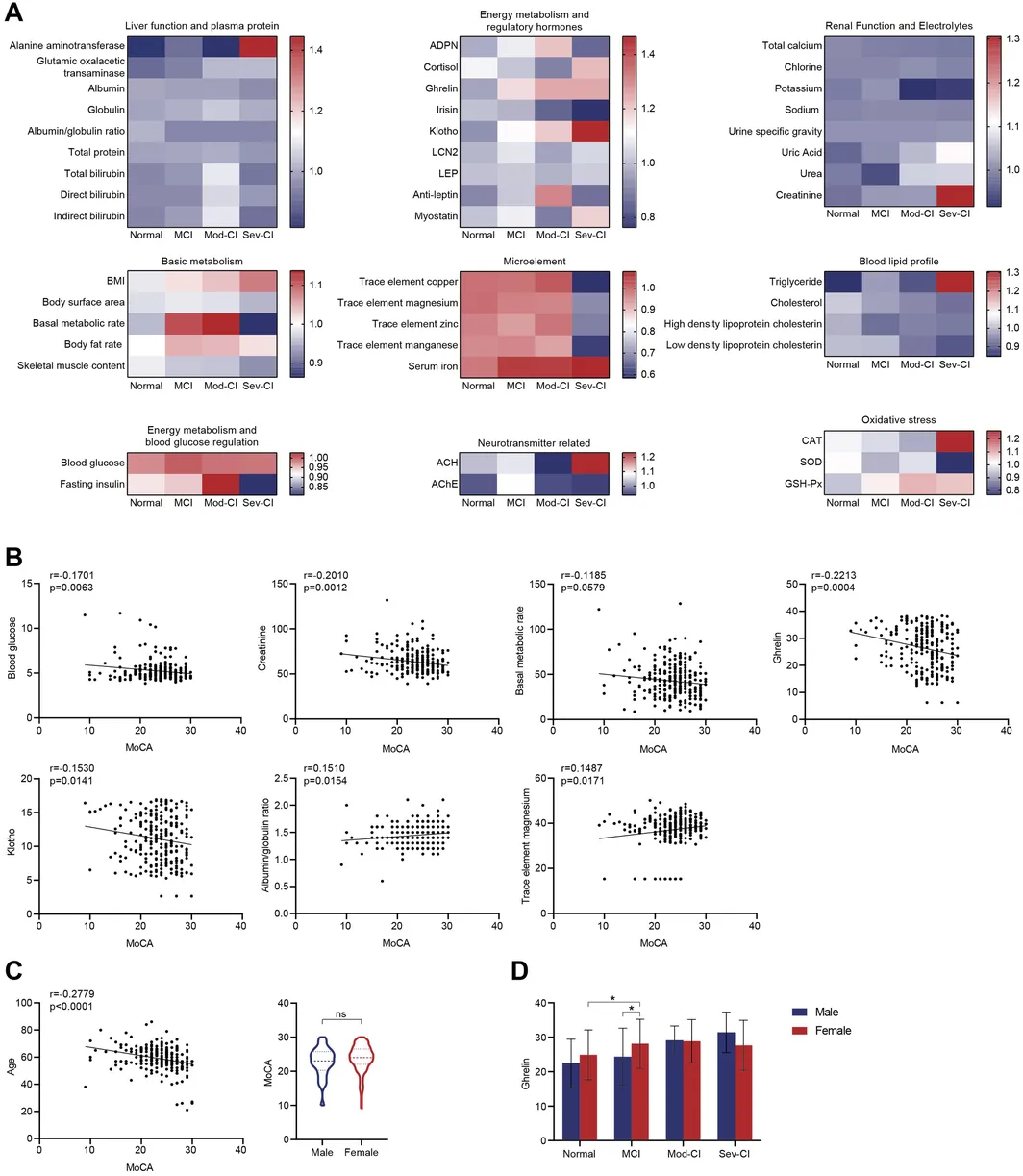
Ghrelin dysregulation is associated with CI in human subjects. (A) Heatmap showing differences in energy metabolism‐related indicators among the Normal, MCI, Mod‐CI, and Sev‐CI groups. (B) Pearson's correlation analysis between MoCA scores and energy metabolism‐related indicators, including blood glucose, creatinine, basal metabolic rate, Ghrelin, Klotho, albumin/globulin ratio, and trace element magnesium. (C) Pearson's correlation analysis between MoCA scores and age, and comparison of MoCA scores between sexes. (D) Differences in ghrelin levels between sexes in the Normal, MCI, Mod‐CI, and Sev‐CI groups. ns, no statistical difference; *p < 0.05.

To further evaluate potential demographic influences, the associations of age and sex with MoCA scores were analyzed. As shown in Figure 1C, age was significantly negatively correlated with MoCA scores, indicating an age‐related decline in cognitive performance, whereas no significant difference in MoCA scores was observed between males and females. The relationship between ghrelin levels and clinical characteristics was further examined. As shown in Table 5, ghrelin levels were not significantly associated with age, sex, years of education, or educational attainment. Consistent with the findings shown in Figure 1B, ghrelin levels remained significantly negatively correlated with MoCA scores.

**TABLE 5:** Correlations of ghrelin with clinical characteristics in participants.

Variables	*r*	*p*
Age (year)	0.02225	0.7344
Sex (male/female)	0.08944	0.1718
Education (years)	0.08420	0.1984
Degree of education	−0.06917	0.2910
MoCA scores	−0.2213	0.0004

Stratified analysis by sex further revealed distinct patterns of ghrelin expression. In males, ghrelin levels did not differ significantly among the four cognitive groups, whereas in females, ghrelin levels were significantly higher in the MCI group than in the cognitively normal group (Figure 1D). Furthermore, females with MCI exhibited significantly higher ghrelin levels than their male counterparts (Figure 1D). These findings suggest that ghrelin may contribute to aging‐related CI in a sex‐dependent manner.

### 3.2 CI in Older and D‐Gal‐Induced Mice Is Accompanied by Microglial Senescence and Elevated Ghrelin Levels

To investigate aging‐related CI and hippocampal senescence, we established an early aging‐related model and a D‐gal‐induced aging model (Figure 2A). Firstly, body weight was monitored, and results showed that young and control mice presented a higher body weight change percentage than older mice and D‐gal‐treated mice (Figure 2B).

**FIGURE 2:**
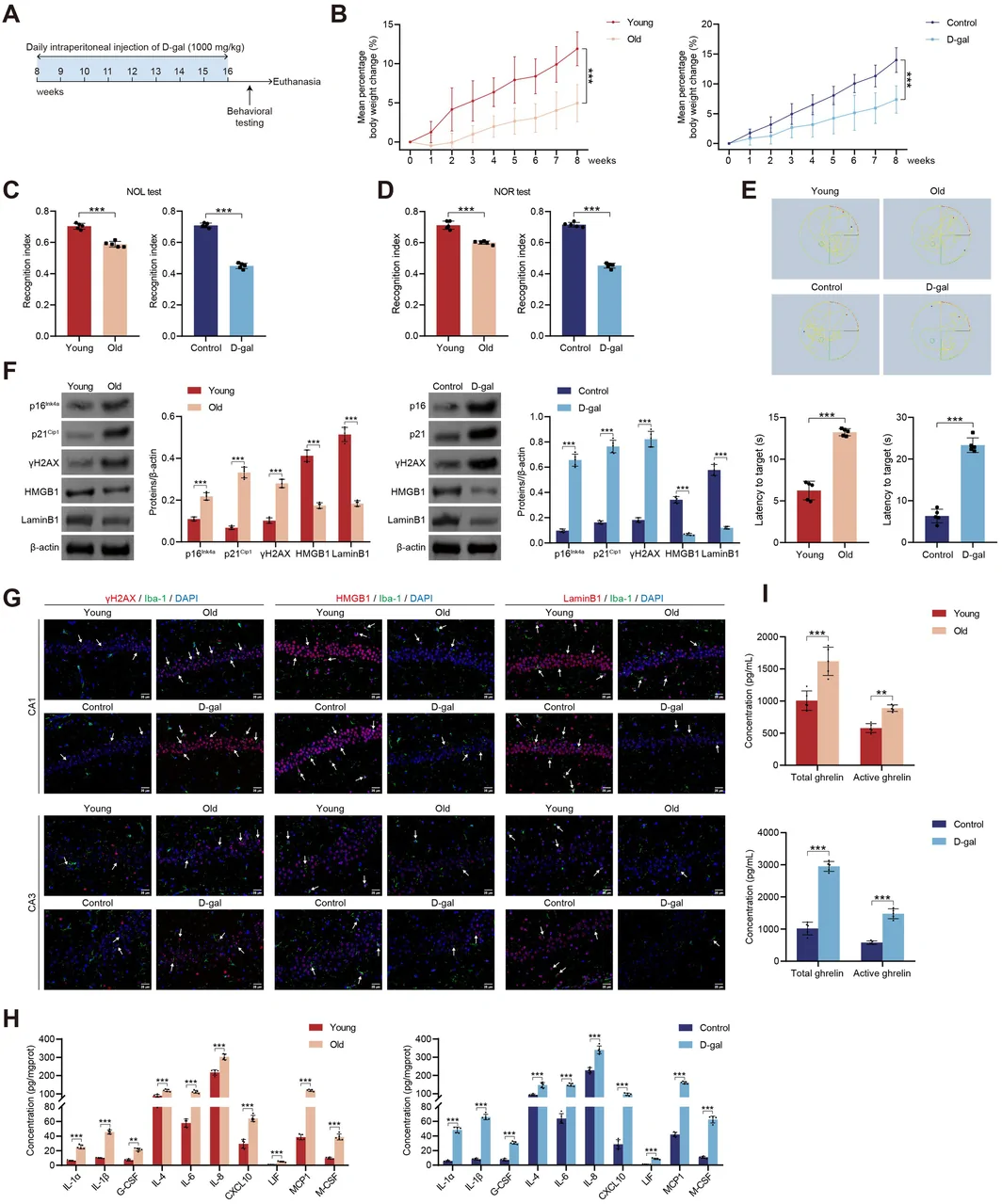
CI in older and D‐gal‐induced mice is accompanied by microglial senescence and elevated ghrelin levels. (A) Experimental design of the D‐gal‐induced aging mouse model. (B) Percentage change in body weight of mice in the Young, Old, Control, and D‐gal groups, normalized to baseline body weight at the start of the experiment. (C) The NOL test comparing the mice from the Young and Old groups and from the Control and D‐gal groups. The recognition index was calculated as the ratio of time spent exploring in the novel location to the total time spent exploring both locations. (D) NOR test comparing the mice from the Young and Old groups and from the Control and D‐gal groups. The recognition index was calculated as the ratio of time spent exploring the novel object relative to the total exploration time for both objects. (E) MWM test comparing the mice from the Young and Old groups and from the Control and D‐gal groups, including swimming trajectories and escape latency. (F) Western blot analysis of senescence markers p16^Ink4a^, p21^Cip1^, LaminB1, γH2AX, and HMGB1 in the mice brain tissue from the Young, Old, Control, and D‐gal groups. (G) Immunofluorescence co‐localization of Iba‐1^+^ microglia with LaminB1, γH2AX, and HMGB1 in the hippocampal CA1 and CA3 regions of the mice from Young, Old, Control, and D‐gal groups (magnification: ×400, scale bar = 25 μm). (H) ELISA‐based quantification of hippocampal cytokines (IL‐1α, IL‐1β, G‐CSF, IL‐4, IL‐6, IL‐8, CXCL10, LIF, MCP1, and M‐CSF) in the Young, Old, Control, and D‐gal groups. (I) ELISA measurement of plasma total ghrelin and acylated ghrelin in the Young, Old, Control, and D‐gal groups. **p < 0.01, ***p < 0.001.

Then, we tested the cognitive ability of mice and found that older mice and D‐gal‐treated mice displayed impaired cognitive ability compared to young and control mice. As shown in Figure 2C,D, older mice and D‐gal‐treated mice presented decreased preference for new objects in the NOL and NOR test, which indicated that older mice and D‐gal‐treated mice presented a deficit in spatial and recognition memory. Meanwhile, in the MWM test, older mice and D‐gal‐treated mice presented increased latency to reach the target platform and impaired spatial learning ability (Figure 2E). Because these behavioral paradigms primarily assess hippocampus‐dependent memory (Drulis‐Fajdasz et al. 2023; Salimi et al. 2020), these findings suggest that aging and D‐gal treatment impair hippocampal function.

Western blot analysis further showed that the expression of senescence‐indicators p16^Ink4a^, p21^Cip1^, and DNA damage marker γH2AX was upregulated, whereas the nuclear envelope protein Lamin B1 and the chromatin‐associated protein HMGB1 were significantly downregulated in the hippocampi of older and D‐gal‐treated mice (Figure 2F). These findings indicate that both early aging and D‐gal treatment induce hippocampal cellular senescence accompanied by cognitive impairment.

Given that cellular senescence within the central nervous system is highly cell type‐specific (Buckley et al. 2023), we next investigated the contribution of microglia to these pathological changes. Microglia are the resident immune cells of the brain parenchyma, and their senescence has been implicated in brain aging and cognitive decline (Costa et al. 2021; Ogrodnik et al. 2021). To verify this possibility, we performed immunofluorescence co‐localization, which showed increased γH2AX and reduced LaminB1 and HMGB1 in Iba‐1^+^ microglia of older and D‐gal mice, suggesting that microglial senescence contributes to aging‐related CI (Figure 2G).

Inflammation is a hallmark of both cognitive impairment and brain aging (Bodles and Barger 2004; Currais et al. 2017; Ogrodnik et al. 2021). Consistent with this observation, ELISA revealed significantly elevated levels of multiple inflammatory mediators, including IL‐1α, IL‐1β, G‐CSF, IL‐4, IL‐6, IL‐8, CXCL10, LIF, MCP‐1, and M‐CSF, in the hippocampi of older and D‐gal‐treated mice compared with their respective controls, consistent with an inflammaging phenotype (Figure 2H). ELISA assays further revealed significantly higher levels of both total ghrelin and acylated ghrelin in older and D‐gal‐treated mice (Figure 2I). Collectively, these results demonstrate that aging and D‐gal treatment are associated with cognitive impairment, microglial senescence, neuroinflammation, and elevated circulating ghrelin levels.

### 3.3 Ghrelin Administration Alleviates D‐Gal‐Induced Microglial Senescence and CI

To investigate the therapeutic effects of ghrelin on aging‐related CI, we first performed a dose–response. D‐gal‐treated mice received low‐ or high‐dose ghrelin during the final 4 weeks of D‐gal administration before behavioral and molecular analyses (Figure S1A). Body weight monitoring showed that D‐gal treatment attenuated body weight gain, whereas ghrelin treatment partially restored this reduction (Figure S1B). Behavioral assessments demonstrated that performance in the NOL, NOR, and MWM tests was significantly improved in ghrelin‐treated mice compared with D‐gal‐treated mice (Figure S1C–E). At the cellular level, immunofluorescence co‐localization revealed that ghrelin treatment decreased the proportion of Iba‐1^+^ microglial cells co‐expressing senescence markers (Figure S1F). Among the treatment groups, high‐dose ghrelin consistently produced the greatest improvements in both behavioral performance and cellular phenotypes and was therefore selected for subsequent experiments.

Next, the protective effects of ghrelin were further evaluated in both the D‐gal‐induced aging model and the early aging‐related model. The experimental design for the D‐gal‐induced aging model is shown in Figure 3A. The same ghrelin dose and treatment regimen were applied to the early aging‐related model. Although body weight increased progressively in all groups, weight gain was significantly reduced in D‐gal‐treated mice compared with control mice and in aged mice compared with young mice. Ghrelin treatment partially restored body weight gain in D‐gal‐treated mice (Figure 3B, Figure S2A).

**FIGURE 3:**
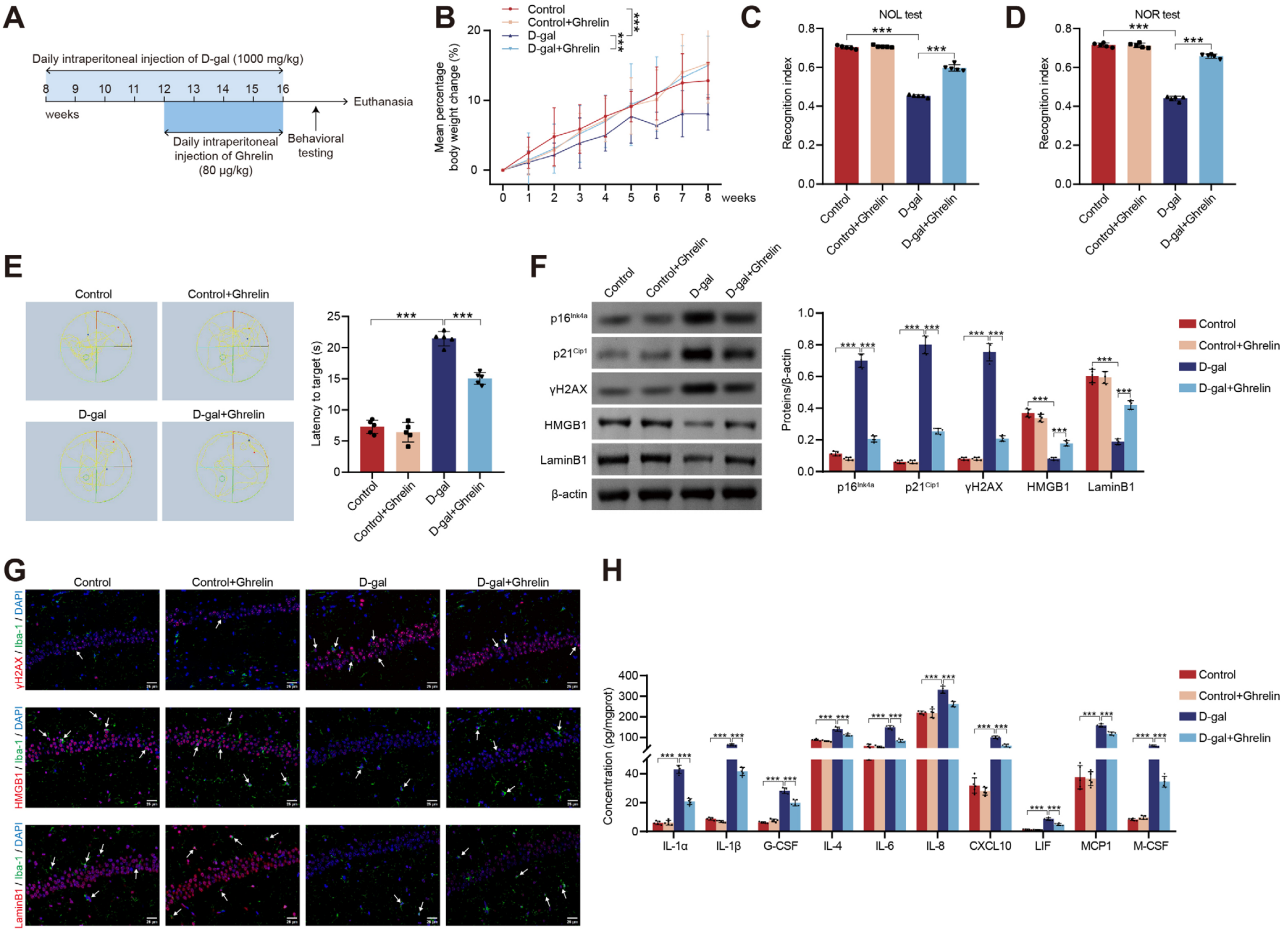
Ghrelin administration alleviates D‐gal‐induced microglial senescence and CI. (A) Experimental design of ghrelin treatment in the D‐gal‐induced aging mouse model. (B) Percentage change in body weight of mice in the Control, Control + Ghrelin, D‐gal, and D‐gal + Ghrelin groups, normalized to baseline body weight at the start of the experiment. (C) The NOL test comparing the mice from the Control, Control + Ghrelin, D‐gal, and D‐gal + Ghrelin groups. The recognition index was calculated as the ratio of time spent exploring in the novel location to the total time spent exploring both locations. (D) NOR test comparing the mice from the Control, Control + Ghrelin, D‐gal, and D‐gal+Ghrelin groups. The recognition index was calculated as the ratio of time spent exploring the novel object relative to the total exploration time for both objects. (E) MWM test comparing the mice from the Control, Control + Ghrelin, D‐gal, and D‐gal + Ghrelin groups, including swimming trajectories and escape latency. (F) Western blot analysis of senescence markers p16^Ink4a^, p21^Cip1^, LaminB1, γH2AX, and HMGB1 in the mice brain tissue from the Control, Control+Ghrelin, D‐gal, and D‐gal+Ghrelin groups. (G) Immunofluorescence co‐localization of Iba‐1^+^ microglia with LaminB1, γH2AX, and HMGB1 in the hippocampal CA1 region of the mice from Control, Control+Ghrelin, D‐gal, and D‐gal + Ghrelin groups (magnification: ×400, scale bar = 25 μm). (H) ELISA‐based quantification of hippocampal cytokines (IL‐1α, IL‐1β, G‐CSF, IL‐4, IL‐6, IL‐8, IP10/CXCL10, LIF, MCP1, and M‐CSF) in the Control, Control + Ghrelin, D‐gal, and D‐gal + Ghrelin groups. ***p < 0.001.

Behavioral testing demonstrated that ghrelin significantly improved performance in the NOL and NOR tests and reduced escape latency in the MWM test in both the D‐gal‐induced and early aging‐related models (Figure 3C–E and Figure S2B–D). Western blot analysis of hippocampal samples revealed that ghrelin markedly downregulated the expression of p16^Ink4a^, p21^Cip1^, and γH2AX while enhancing the expression of LaminB1 and HMGB1, suggesting reversal of the senescence phenotype (Figure 3F, Figure S2E). Further immunofluorescence co‐localization analysis in the D‐gal model also demonstrated that ghrelin decreased the number of Iba‐1^+^ microglia co‐expressing γH2AX and further rescued the co‐localization of LaminB1 and HMGB1 with Iba‐1 (Figure 3G). Moreover, the co‐localization of γH2AX with NeuN^+^ neurons was also increased in D‐gal‐treated mice and reduced after ghrelin administration, whereas γH2AX showed minimal co‐localization with GFAP^+^ astrocytes (Figure S3A). In addition, ELISA assays demonstrated that ghrelin reduced the levels of multiple pro‐inflammatory cytokines in the hippocampus compared with the D‐gal group (Figure 3H). Taken together, our results demonstrated that ghrelin could ameliorate cognitive and cellular deficits in D‐gal‐induced aging and early aging‐related models.

### 3.4 Network Pharmacology and Molecular Docking Suggest SHBG as a Candidate Mediator of Ghrelin Action

To investigate the mechanisms underlying the protective effects of ghrelin, we first examined whether high‐dose ghrelin altered GHSR function. GHSR is the receptor for ghrelin and has been implicated in aging and cognitive impairment. Membrane localization of GHSR reflects receptor activation, whereas receptor internalization is indicative of desensitization (Tian, Wang, and Du 2023). Western blot analysis showed that restoration of GHSR membrane localization was more pronounced in the low‐dose ghrelin group than in the high‐dose group. Consistently, only low‐dose ghrelin increased the p‐CaMKII/CaMKII ratio, a downstream indicator of GHSR‐mediated synaptic plasticity, compared with the D‐gal group (Figure S3B). These findings suggest that high‐dose ghrelin may induce GHSR desensitization (Kern et al. 2015; Tian, Du, et al. 2023).

To further determine whether the protective effects of ghrelin depend on GHSR signaling, D‐gal‐treated primary microglia were treated with ghrelin following GHSR knockdown. GHSR knockdown did not significantly affect the ghrelin‐mediated downregulation of p16^Ink4a^, p21^Cip1^, or γH2AX, but partially weakened the recovery of HMGB1 and LaminB1 (Figure S3C). These results suggest that, although some protective effects of high‐dose ghrelin may involve GHSR signaling, its anti‐senescent activity is largely GHSR‐independent.

To identify candidate downstream mediators of the anti‐senescent effects of ghrelin, network pharmacology analysis was performed. Venn diagram analysis showed that ghrelin and CI had 202 common targets (Figure 4A). A PPI network was constructed using the targets, and then MCODE clustering identified six gene clusters. Five hub genes, SHBG, SRC, C5AR1, KLF4, and BRCA1, were subsequently identified as candidate mediators that may contribute to the protective effects of ghrelin against CI (Figure 4B,C).

**FIGURE 4:**
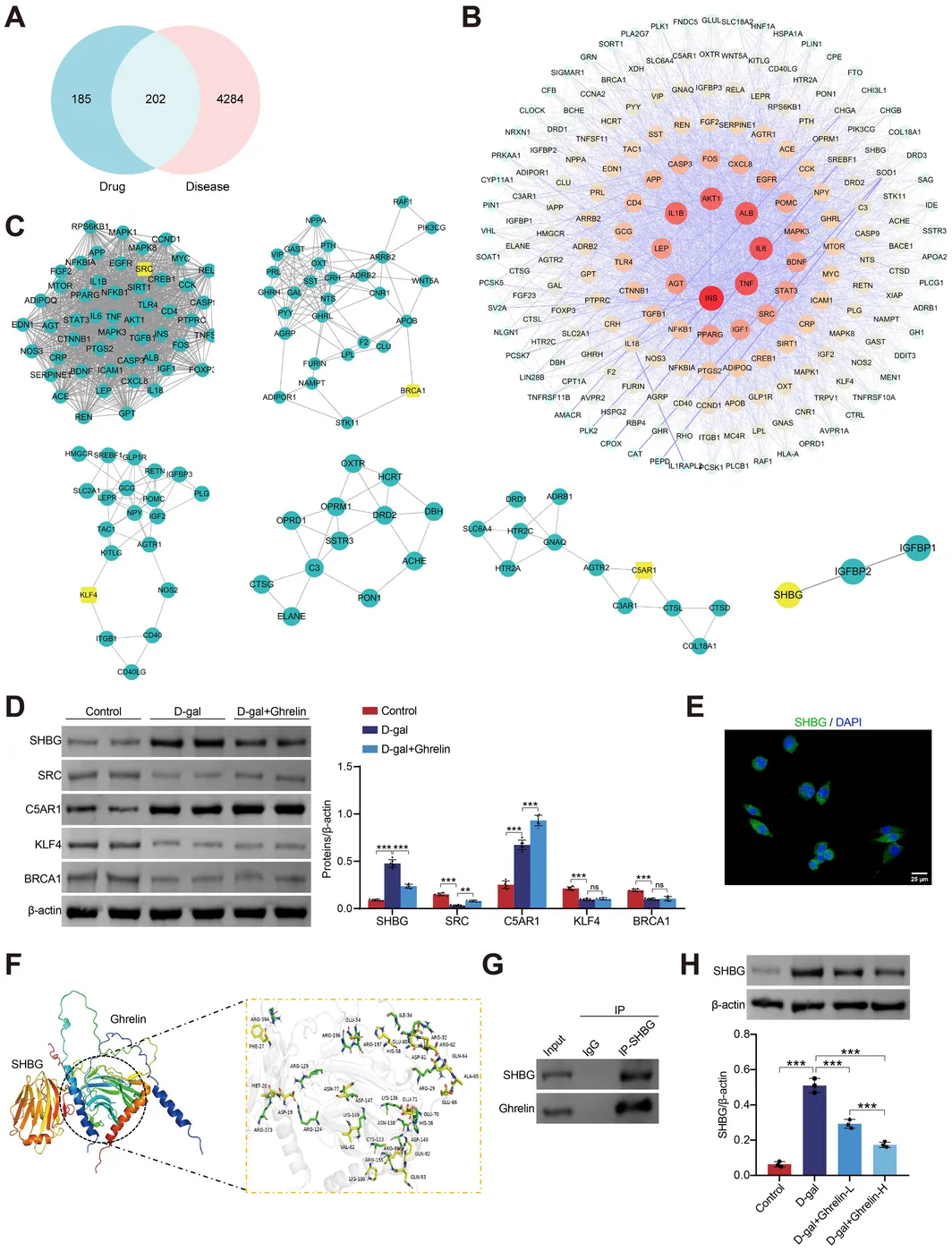
Network pharmacology and molecular docking suggest SHBG as a candidate mediator of ghrelin action. (A) Venn diagram showing the overlap between predicted ghrelin‐ and CI‐related targets. (B) PPI network of shared drug‐disease targets. (C) MCODE clustering analysis of the drug‐disease targets, identifying gene clusters and core genes. (D) Western blot detection of SHBG, SRC, C5AR1, KLF4, and BRCA1 expression in hippocampal tissue from mice in the control, D‐gal, and D‐gal + Ghrelin groups. (E) Subcellular localization of SHBG in BV2 cells identified by immunofluorescence (magnification: ×400, scale bar = 25 μm). (F) Molecular docking analysis of ghrelin with human SHBG. (G) Co‐IP assay validating the interaction between ghrelin and SHBG in primary microglia. (H) Western blot analysis of SHBG expression in hippocampal tissue from mice in the Control, D‐gal, D‐gal + Ghrelin‐L, and D‐gal + Ghrelin‐H groups. ns, *p* > 0.05, **p < 0.01, ***p < 0.001.

We next explored whether these candidate molecules were regulated in vivo. Compared with the control group, D‐gal treatment increased the expression of SHBG and C5AR1, while decreasing the expression of SRC, KLF4, and BRCA1. Among these genes, SHBG exhibited the most consistent response to ghrelin treatment, with its D‐gal‐induced upregulation being partially reversed. In contrast, SRC and C5AR1 were increased following ghrelin treatment, whereas KLF4 and BRCA1 showed minimal changes (Figure 4D). Previous studies have demonstrated that increased plasma SHBG levels are associated with accelerated hippocampal atrophy and cognitive decline (Xu et al. 2020). Therefore, SHBG might be associated with pathogenic processes in aging‐induced CI.

Immunofluorescence staining showed that SHBG was predominantly localized in the cytoplasm of BV2 cells (Figure 4E). Molecular docking analysis suggested a potential interaction between ghrelin and SHBG, which was further supported by co‐IP experiments in primary microglia (Figure 4F,G). Finally, Western blot analysis demonstrated that D‐gal treatment significantly increased hippocampal SHBG expression, whereas ghrelin reduced SHBG levels in a dose‐dependent manner, with the high‐dose treatment producing a greater effect than the low‐dose treatment (Figure 4H). Taken together, our data suggested that although high doses of ghrelin may induce GHSR desensitization, SHBG may serve as a potential mediator contributing to the protective effects of ghrelin against CI.

### 3.5 Ghrelin Regulates Aging‐Related CI Through Autophagy‐Related Signaling in Microglia

KEGG pathway enrichment analysis found that cellular senescence and autophagy‐related pathways were significantly enriched as major pathways related to ghrelin (Figure 5A). GO enrichment analysis further identified SHBG as a significantly enriched molecule associated with hormone binding (Figure 5A). To experimentally validate these bioinformatic predictions in vivo, hippocampal tissues from D‐gal‐treated mice with or without ghrelin treatment were analyzed. RT‐qPCR analysis showed that ghrelin downregulated the expression of AKT1, CTSL, ERK1, and LKB1 while upregulating MTOR, RAF1, and JNK1, with JNK1 exhibiting the most pronounced change (Figure 5B). Western blot analyses demonstrated that ghrelin altered the autophagy‐related markers, decreasing the levels of p62 and increasing the levels of Beclin1 and LC3‐II/LC3‐I (Figure 5C). Immunofluorescence staining analysis further demonstrated that ghrelin decreased the co‐localization of p62 and γH2AX in Iba‐1^+^ microglia, while enhancing the co‐localization of p62 and LaminB1 in Iba‐1^+^ cells, which suggested that ghrelin relieved the senescence of microglia, possibly through autophagy‐associated molecular changes (Figure 5D).

**FIGURE 5:**
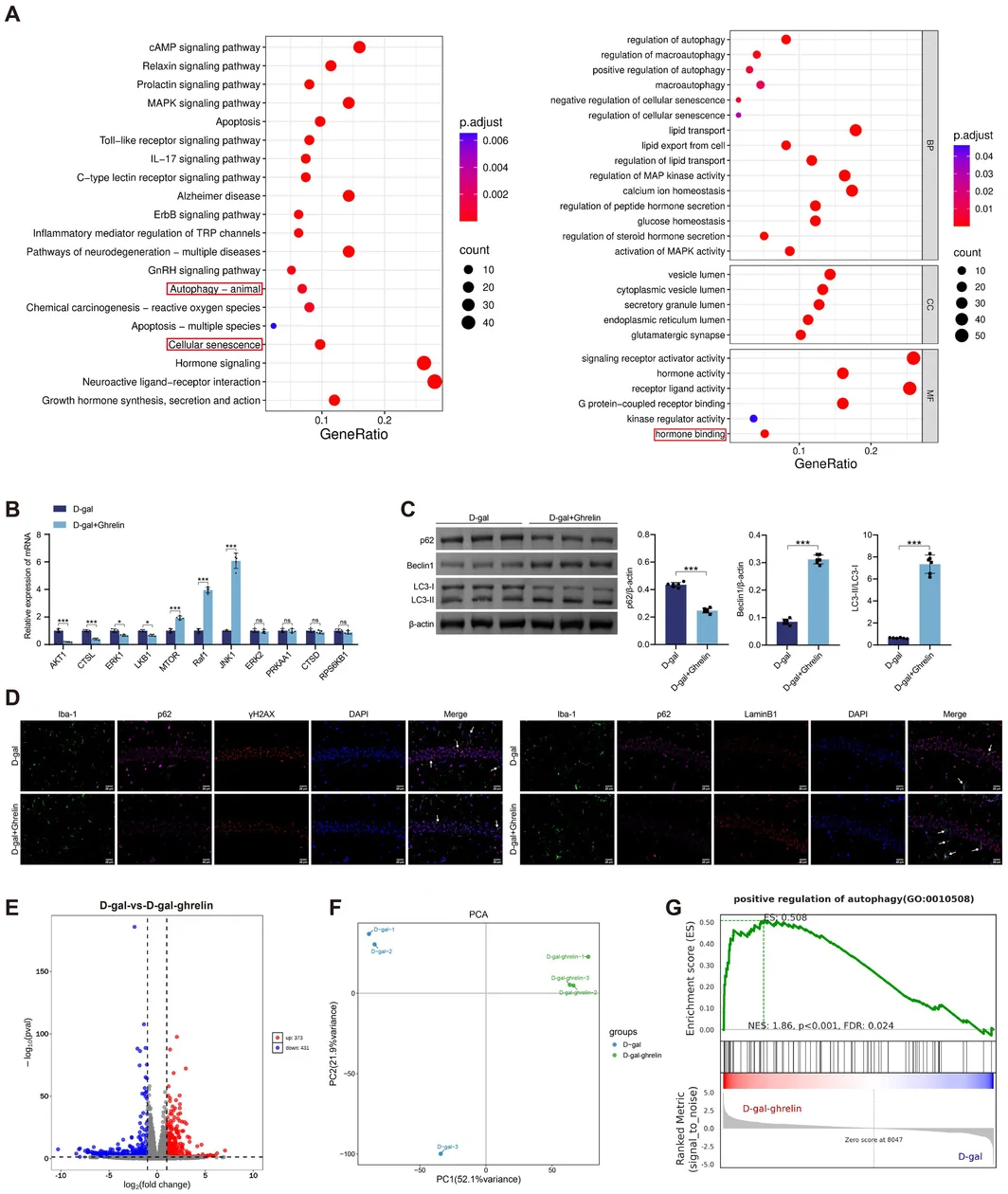
Ghrelin regulates aging‐related CI through autophagy‐related signaling in microglia. (A) KEGG and GO enrichment analyses of predicted ghrelin‐ and CI‐related targets. (B) RT‐qPCR detection of autophagy‐related genes (AKT1, CTSL, ERK1, LKB1, MTOR, RAF1, JNK1, ERK2, PRKAA1, CTSD, and RPS6KB1) in hippocampal tissue from mice treated with D‐gal or D‐gal + Ghrelin for 4 weeks. (C) Western blot analysis of autophagy‐related markers p62, Beclin1, and LC3 in hippocampal tissue from mice in the D‐gal and D‐gal + Ghrelin groups. (D) Immunofluorescence staining of Iba‐1^+^ microglia co‐localized with autophagy‐related marker p62 and senescence markers LaminB1 and γH2AX in the hippocampal CA1 region of mice in the D‐gal and D‐gal + Ghrelin groups (magnification: ×400, scale bar = 25 μm). (E) Volcano plot visualization of differentially expressed genes in primary microglia treated with D‐gal or D‐gal + Ghrelin. (F) PCA of transcriptomic differences between D‐gal and D‐gal + Ghrelin groups. (G) GSEA functional enrichment analysis in primary microglia. ns, *p* > 0.05, *p < 0.05, ***p < 0.001.

To determine the optimal duration and concentration of ghrelin treatment in vitro, primary microglia were first treated with D‐gal and subsequently exposed to ghrelin for 30 min, 3 h, or 18 h or at concentrations of 0.1, 1, or 10 μM. Ghrelin treatment at 1 μM for 30 min produced the most pronounced anti‐senescent effects (Figure S4A,B). RNA‐seq was subsequently performed on primary microglia treated with D‐gal in the presence or absence of ghrelin. Using the thresholds of |log_2_(fold change)| > 1 and adjusted *p* < 0.05, Volcano plot analysis identified 373 upregulated and 431 downregulated genes in ghrelin‐treated cells relative to D‐gal‐treated cells (Figure 5E), and PCA demonstrated clear separation between the two groups (Figure 5F). GSEA analysis indicated positive enrichment of autophagy‐related pathways in ghrelin‐treated microglia with normalized enrichment scores (NES) of 1.86 (Figure 5G). Collectively, our results demonstrated that ghrelin may alleviate microglial senescence by enhancing autophagy‐related mechanisms.

### 3.6 Ghrelin Alleviates Microglial Senescence Through Modulating Autophagy‐Related Signaling via the SHBG/JNK1/Beclin1 Axis

Next, we investigated whether SHBG is involved in the autophagy‐related effects of ghrelin. We detected SHBG and autophagy‐related markers in microglial cells. Ghrelin decreased SHBG and p62 and enhanced Beclin1 and LC3‐II/LC3‐I (Figure 6A). Overexpression of SHBG partially abolished the effects of ghrelin. At the level of senescence‐associated proteins, overexpressing SHBG increased the expression of p21^Cip1^ and γH2AX and reduced the expression of LaminB1 and HMGB1, indicating that the senescent phenotype was aggravated (Figure 6B). Consistently, SHBG overexpression altered the expression of autophagy‐related signaling molecules by increasing p‐Raf1 while decreasing Beclin1, the LC3‐II/LC3‐I ratio, and the p‐JNK1/JNK1 ratio (Figure 6B,C).

**FIGURE 6:**
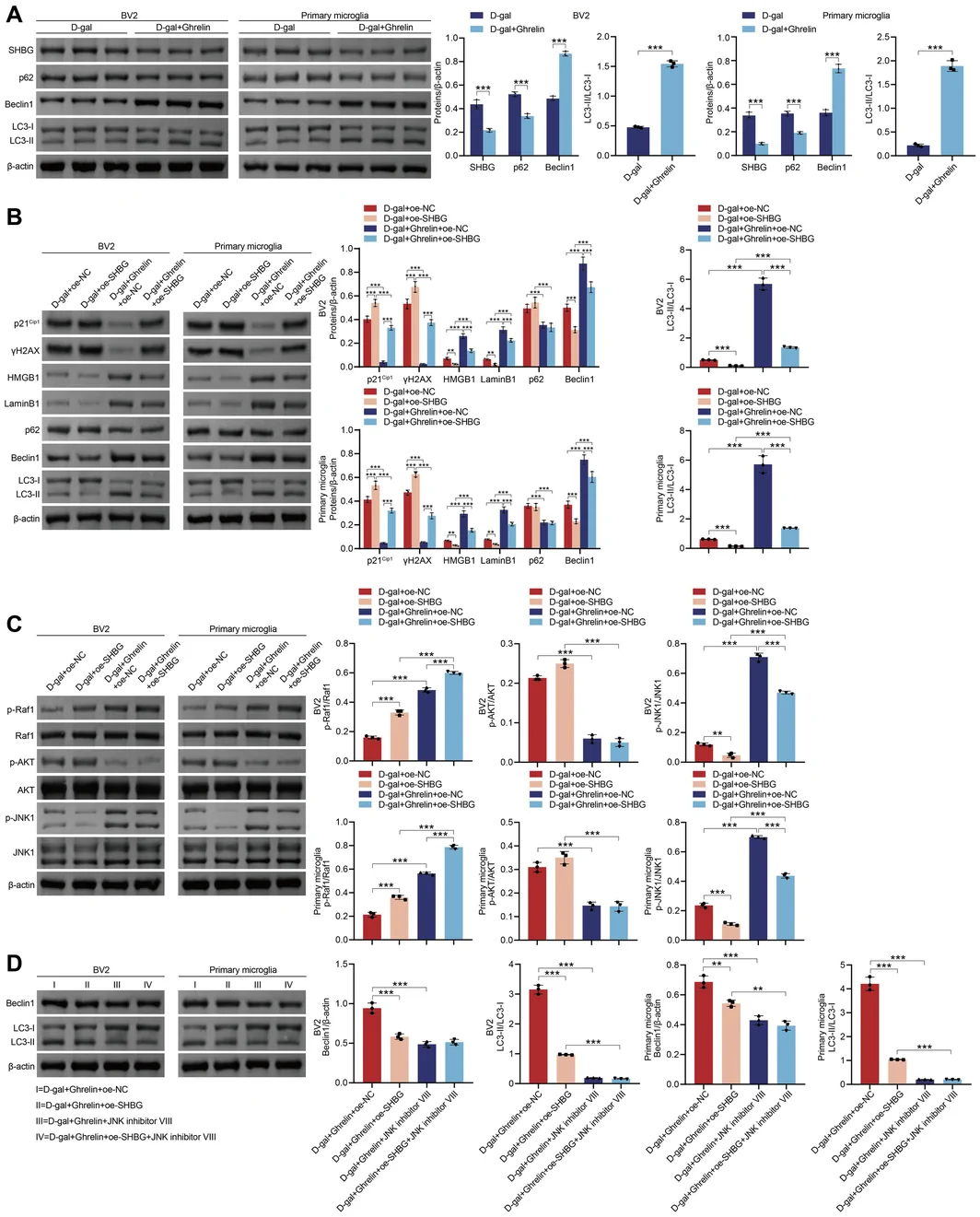
Ghrelin alleviates microglial senescence by modulating autophagy‐related signaling via the SHBG/JNK1/Beclin1 axis. (A) Western blot analysis of SHBG and autophagy‐related proteins (p62, Beclin1, and LC3) in BV2 cells and primary microglia exposed to D‐gal or D‐gal + Ghrelin. (B) Western blot analysis of autophagy‐related proteins (p62, Beclin1, and LC3) and senescence‐related proteins (p21^Cip1^, LaminB1, γH2AX, and HMGB1) in BV2 cells and primary microglia from the D‐gal + oe‐NC, D‐gal + oe‐SHBG, D‐gal + Ghrelin + oe‐NC, and D‐gal + Ghrelin + oe‐SHBG groups. (C) Western blot analysis of p‐Raf1/Raf1, p‐AKT/AKT, and p‐JNK1/JNK1 ratios in BV2 cells and primary microglia from the D‐gal + oe‐NC, D‐gal + oe‐SHBG, D‐gal + Ghrelin + oe‐NC, and D‐gal + Ghrelin + oe‐SHBG groups. (D) Western blot analysis of Beclin1 and LC3 in BV2 cells and primary microglia from the D‐gal + Ghrelin + oe‐NC, D‐gal + Ghrelin + oe‐SHBG, D‐gal + Ghrelin + oe‐NC + JNK inhibitor VIII, and D‐gal+Ghrelin + oe‐SHBG + JNK inhibitor VIII groups. **p < 0.01, ***p < 0.001.

To further validate the regulatory relationship between SHBG and JNK1, SHBG expression was manipulated in BV2 microglial cells (Figure S5A). SHBG knockdown increased JNK1 phosphorylation, whereas SHBG overexpression reduced JNK1 phosphorylation (Figure S5B), supporting that JNK1 may act downstream of SHBG. Pharmacological inhibition of JNK1 activity markedly reduced Beclin1 levels and LC3‐II/LC3‐I under ghrelin treatment (Figure 6D).

To elucidate whether Beclin1 functions downstream of SHBG/JNK1 signaling, we co‐overexpressed Beclin1 in the condition of SHBG overexpression and treated D‐gal‐exposed cells with ghrelin. Beclin1 overexpression partially rescued the effects of SHBG overexpression on senescence‐associated proteins, reversing the suppression of ghrelin‐mediated protection (Figure S5C), indicating that Beclin1 is involved in mediating ghrelin's anti‐senescent effects. These results suggested that ghrelin alleviated the senescence of microglia, possibly involving autophagy‐related processes through the SHBG/JNK1/Beclin1 signaling axis.

## 4 Discussion

The present study demonstrates that ghrelin alleviates aging‐related CI by attenuating microglial senescence and modulating autophagy‐related molecular changes through the SHBG/JNK1/Beclin1 signaling axis. Cohort analysis identified ghrelin as the metabolic factor most strongly associated with CI. In both the D‐gal‐induced and early aging‐related models, ghrelin improved learning and memory, suppressed hippocampal inflammatory cytokine production, and attenuated the expression of senescence‐associated markers. The desensitization of GHSR under high‐dose ghrelin exposure raises the possibility that SHBG may serve as a potential non‐canonical mediator of ghrelin's anti‐senescent effects. Mechanistically, ghrelin modulated autophagy‐related signaling through the SHBG/JNK1/Beclin1 axis, thereby attenuating microglial senescence. This study improves our understanding of metabolic signaling in immunosenescence, providing a theoretical basis for the application of ghrelin as a potential intervention target for aging‐related CI.

Cognitive decline in aging is closely associated with immune dysfunction and chronic inflammation (Li et al. 2025; Mohapatra et al. 2023). Immunosenescence facilitates the accumulation of dysfunctional T cells, aberrant neuroimmune modulation, and increased proinflammatory cytokine release, which further promote neurodegeneration (Li et al. 2025; Mohapatra et al. 2023). Both experimental and clinical studies have demonstrated that aging‐related immune dysfunction promotes neuronal injury and cognitive decline (Li et al. 2022; Mohapatra et al. 2023). Similarly, persistent activation of inflammatory signaling pathways in CI has also been extensively reported as a typical feature of CI (Arnoriaga‐Rodriguez and Fernandez‐Real 2019; Childs et al. 2024; Ruiz‐Fernandez et al. 2024). Meta‐analysis results demonstrated that the levels of pro‐inflammatory cytokines such as IL‐1β, IL‐6, IL‐8, and CRP were markedly up‐regulated in MCI and Alzheimer's patients compared with the healthy group, and these changes were sex‐ and age‐dependent (Childs et al. 2024). Our results showed that the hippocampal cytokines were upregulated in older and D‐gal‐treated mice, which was consistent with the above literature, indicating that immunosenescence and inflammation together promote cognitive impairment.

As a gut‐derived peptide, ghrelin has been proposed to be involved in neuroinflammation and microglial function in the regulation of cognitive function (Russo et al. 2022). Signaling of ghrelin through GHSR modulates microglial polarization and inhibits the production of proinflammatory cytokines, and excessive or chronic stimulation can induce GHSR desensitization (Shen et al. 2025). Similarly, our results revealed that older and D‐gal mice exhibited elevated circulating ghrelin levels, and high‐dose ghrelin was associated with less GHSR membrane localization than low‐dose, suggesting receptor desensitization. The elevated ghrelin levels observed in human subjects may reflect a compensatory response or serve as a metabolic stress‐related biomarker, rather than indicating a causal detrimental role, thereby reconciling the association between higher circulating ghrelin levels and poorer cognitive performance in the human cohort with the protective effects of exogenous ghrelin treatment observed in the animal models. Interestingly, ghrelin has also been reported to regulate autophagy, an essential process for maintaining microglial homeostasis (Chung et al. 2018). It has been demonstrated previously that ghrelin prevents the suppression of autophagic homeostasis by inhibiting excessive autophagy‐induced cell death during stress while maintaining autophagy homeostasis (Chung et al. 2018; Reich and Holscher 2020). Furthermore, because ghrelin possesses both anti‐inflammatory and autophagy‐regulating properties, it has been proposed as a potential multi‐target therapeutic agent for Alzheimer's and Parkinson's diseases (Reich and Holscher 2020). However, large‐scale cohort analyses (van Andel et al. 2021) and meta‐analyses (Garcia‐Garcia et al. 2023) have not found a consistent relationship between ghrelin and cognitive decline, highlighting the need for mechanistic studies.

Our results showed that ghrelin treatment attenuated the expression of microglial senescence markers and increased Beclin1 and LC3‐II levels, indicating improved autophagy‐related molecular changes, consistent with its well‐known protective effects (Chung et al. 2018; Reich and Holscher 2020). In addition, ghrelin reduced γH2AX signals in neurons from D‐gal‐treated mice, suggesting that its protective effects may not be limited to microglia. Given that microglia are the primary innate immune cells of the central nervous system and are closely associated with neuroinflammation and autophagy dysregulation (Colonna and Butovsky 2017), our mechanistic investigations primarily focused on microglia.

Network pharmacology and validation experiments suggested SHBG as a candidate mediator of ghrelin action, and their interaction may occur in the cytoplasm of microglia. Clinical and biomarker studies reported that elevated SHBG levels were associated with cognitive decline in schizophrenia and frontotemporal lobar degeneration patients (Campo et al. 2021; Xu et al. 2024). Consistent with our study, SHBG was involved in the pathogenesis of schizophrenia by impairing neuroprotection. SHBG may function as a negative regulator of neuroprotection, possibly through sequestration of hormones or modulating receptor availability (Campo et al. 2021; Xu et al. 2024). Mechanistically, we found that the alteration of autophagy‐related molecules by ghrelin was dependent on JNK1‐mediated activation of Beclin1. Previous reports indicated that JNK1 phosphorylates Bcl‐2 to release Beclin1 and activate autophagy (Ke et al. 2019; Zhou et al. 2015). In our experiments, SHBG overexpression decreased the levels of Beclin1 and LC3 and enhanced senescence markers, while JNK1 inhibition abolished the effect of ghrelin on promoting autophagy‐related signaling. Importantly, co‐treatment with SHBG overexpression and JNK1 inhibition did not produce additive effects compared with JNK1 inhibition alone (Figure 6D). We proposed and demonstrated that SHBG is localized upstream of JNK1, and that when JNK1 was inhibited, SHBG could not provide additional suppression. Our results were consistent with the canonical JNK1‐Beclin1 axis involved in autophagy regulation (Ke et al. 2019; Zhou et al. 2015).

Interestingly, we found that the expression of some autophagy‐related genes (ERK2, PRKAA1, CTSD, and RPS6KB1) was not significantly altered by ghrelin treatment (Figure 5B). This phenomenon might be related to compensatory effects in the autophagy‐related regulatory network. It has been reported that ghrelin preferentially activates JNK1 and RAF1 (Russo et al. 2022; Shen et al. 2025), while ERK and AMPK were not the main targets in our experimental conditions. Lysosomal proteases (CTSD) and ribosomal kinase (RPS6KB1) may have acted downstream of the major events initiating autophagy and thus were not affected by ghrelin treatment in the short term (Clark et al. 2024; DeAguero et al. 2025). These results demonstrate the pathway‐specific effect of ghrelin and suggest that not all autophagy‐related regulators are modulated by ghrelin.

There are several limitations in the present study. Firstly, we employed early aging‐related and D‐gal models to induce CI, but these models cannot completely recapitulate the complex pathology of human CI. In addition, the mice used in the early aging‐related model were 9 months old or older, which may more closely represent a middle‐aged or early aging model rather than a classical advanced aging model. Meanwhile, the animal experiments in this study were conducted in male mice, so the applicability of the findings to female individuals needs to be further verified. Secondly, we provided evidence supporting the involvement of the SHBG/JNK1/Beclin1 signaling pathway in microglia, but whether microglia are associated with other signaling pathways, such as AMPK or ERK, remains to be investigated. Lastly, the long‐term effect and safety of ghrelin treatment should be evaluated in large animal models and clinical trials.

Taken together, these findings identify ghrelin as a novel modulator of aging‐related CI. Ghrelin may ameliorate microglial senescence by modulating autophagy‐related signaling through the SHBG/JNK1/Beclin1 pathway, which may partly contribute to the suppression of inflammation and improvement of cognitive function. This study provides new insights into the metabolic regulation of brain aging and provides a potential therapeutic for aging‐related CI.

## Author Contributions

L.Z., Y.C. and X.L. contributed to the design, collection, analysis, and description of data. W.Z., L.Z., X.S., F.H., Y.Z., M.G. and G.Z. were involved in data acquisition and organization. L.Z., Y.C. and B.D. performed the analysis and wrote the paper. X.L. revised the manuscript critically and supervised the study. All authors discussed and revised the manuscript, ultimately reviewing and approving the final submitted version.

## Funding

This work was supported by the Natural Science Foundation of Sichuan Province, China (2024NSFSC1604, 2025ZNSFSC1597), the National Clinical Research Center for Geriatrics, West China Hospital, Sichuan University (Z2023LC005, Z2024JC008), the Project of Sichuan Provincial Health Commission (24LCYJPT01), Project of Chengdu Science and Technology (2024‐YF05‐02255‐SN), and 1·3·5 project for disciplines of excellence—Clinical Research Fund, West China Hospital, Sichuan University (2025HXFH040).

## Ethics Statement

This study was approved by the Ethics Committee on Biomedical Research, West China Hospital of Sichuan University (No. 2020‐287). Informed consent was obtained from the participants. The patients agreed to participate in our study. They decided to use their data for our study. All the data and material have been performed following the Declaration of Helsinki and conformed to relevant aspects of the ARRIVE guidelines. Animal experiments were approved by the Animal Ethics Committee of West China Hospital, Sichuan University (No. 20240221006).

## Consent

The authors have nothing to report.

## Conflicts of Interest

The authors declare no conflicts of interest.

## Supporting information


**Figure S1:** Screening of ghrelin intervention dosage in D‐gal‐induced aging mice.
**Figure S2:** Ghrelin administration alleviates senescence and CI in an early aging‐related model.
**Figure S3:** High‐dose ghrelin intervention and assessment of GHSR desensitization in D‐gal‐treated mice.
**Figure S4:** Screening of Ghrelin intervention time and dosage.
**Figure S5:** Validation of the SHBG/JNK1/Beclin1 axis in BV2 microglia.

## Data Availability

The data used for the network pharmacology analysis in this study were obtained from publicly available databases, including PubChem (https://pubchem.ncbi.nlm.nih.gov/), the Similarity Ensemble Approach (SEA) database (https://sea.bkslab.org/), the Comparative Toxicogenomics Database (CTD; https://ctdbase.org/), GeneCards (https://www.genecards.org/), UniProt (https://www.uniprot.org/), the National Center for Biotechnology Information (NCBI; https://www.ncbi.nlm.nih.gov/), the Online Mendelian Inheritance in Man (OMIM) database (https://www.omim.org/), Venny 2.1 (https://bioinfogp.cnb.csic.es/tools/venny/), and STRING (https://string‐db.org/). The RNA sequencing data generated in this study have been deposited in the NCBI Sequence Read Archive (SRA; https://www.ncbi.nlm.nih.gov/sra/) under BioProject accession number PRJNA1522985.
