# Supplementary material for: Ghrelin Alleviates Aging‐Related Cognitive Impairment by Regulating Autophagy‐Related Signaling via the SHBG/JNK1/Beclin1 Axis in Microglia
Source: Aging Cell. 2026 Sep 29;25(10):e70732. doi: 10.1111/acel.70732 (PMC13621234; doi:10.1111/acel.70732)
Supplement: Supplementary file 1 [file ACEL-25-e70732-s001.docx]

**Supplementary Information**

**Supplementary Figure 1. Screening of ghrelin intervention dosage in D-gal-induced aging mice.** (A) Experimental design of low- and high-dose ghrelin treatment in the D-gal-induced aging mouse model. (B) Percentage change in body weight of mice in the Control, D-gal, D-gal+Ghrelin-L, and D-gal+Ghrelin-H groups, normalized to baseline body weight at the start of the experiment. (C) NOL test comparing the mice from the Control, D-gal, D-gal+Ghrelin-L, and D-gal+Ghrelin-H groups. The recognition index was calculated as the ratio of time spent exploring in the novel location to the total time spent exploring both locations. (D) NOR test comparing the mice from the Control, D-gal, D-gal+Ghrelin-L, and D-gal+Ghrelin-H groups. The recognition index was calculated as the ratio of time spent exploring the novel object relative to the total exploration time for both objects. (E) MWM test comparing the mice from the Control, D-gal, D-gal+Ghrelin-L, and D-gal+Ghrelin-H groups, including swimming trajectories and escape latency. (F) Immunofluorescence co-localization of Iba-1^+^ microglia with senescence markers p16^Ink4a^, p21^Cip1^, and γH2AX in the hippocampal CA1 region of the mice from the Control, D-gal, D-gal+Ghrelin-L, and D-gal+Ghrelin-H groups (magnification: ×400, scale bar = 25 μm). *p<0.05, **p<0.01, ***p<0.001.

**Supplementary Figure 2. Ghrelin administration alleviates senescence and CI in an early aging-related model.** (A) Percentage change in body weight of mice in the Young, Old, and Old+Ghrelin groups, normalized to baseline body weight at the start of the experiment. (B) NOL test comparing the mice from the Young, Old, and Old+Ghrelin groups. The recognition index was calculated as the ratio of time spent exploring in the novel location to the total time spent exploring both locations. (C) NOR test comparing the mice from the Young, Old, and Old+Ghrelin groups. The recognition index was calculated as the ratio of time spent exploring the novel object relative to the total exploration time for both objects. (D) MWM test comparing the mice from the Young, Old, and Old+Ghrelin groups, including swimming trajectories and escape latency. (E) Western blot analysis of senescence markers p16^Ink4a^, p21^Cip1^, LaminB1, γH2AX, and HMGB1 in the mice's brain tissue from the Young, Old, and Old+Ghrelin groups. *P < 0.05, **P < 0.01, ***P < 0.001.

**Supplementary Figure 3. High-dose ghrelin intervention and assessment of GHSR desensitization in D-gal-treated mice.** (A) Immunofluorescence co-localization of senescence marker γH2AX with NeuN^+^ neurons and GFAP^+^ astrocytes in the hippocampal CA1 region of the mice from Control, Control+Ghrelin, D-gal, and D-gal+Ghrelin groups (magnification: ×400, scale bar = 25 μm). (B) Western blot analysis of hippocampal GHSR distribution and p-CaMKII/CaMKII ratio in the mouse brain tissue from D-gal, D-gal+Ghrelin-L, and D-gal+Ghrelin-H groups. (C) Western blot analysis of senescence markers p16^Ink4a^, p21^Cip1^, LaminB1, γH2AX, and HMGB1 in primary microglial cells from Control, D-gal, D-gal+Ghrelin, D-gal+Ghrelin+sh-NC, and D-gal+Ghrelin+sh-GHSR groups. ns, not significant, **p<0.01, ***p<0.001.

**Supplementary Figure 4. Screening of Ghrelin intervention time and dosage.** (A) Western blot analysis of senescence markers p16^Ink4a^, p21^Cip1^, LaminB1, γH2AX, and HMGB1 in primary microglia treated with D-gal (250 mM, 24 h) and ghrelin (1 μM) for 30 min, 3 h, or 18 h, compared with untreated control and D-gal groups. (B) Western blot analysis of the same senescence markers in primary microglia treated with D-gal and ghrelin at different concentrations (0.1, 1, and 10 μM) for 30 min, compared with control and D-gal groups. **p<0.01, ***p<0.001.

**Supplementary Figure 5. Validation of the SHBG/JNK1/Beclin1 axis in BV2 microglia.** (A) RT-qPCR validation of knockdown and overexpression efficiency of SHBG (sh-SHBG and oe-SHBG). (B) Western blot analysis of JNK1 phosphorylation levels in BV2 cells from sh-sssNC, sh-SHBG#1, sh-SHBG#2, oe-NC, and oe-SHBG groups. (C) Western blot analysis of senescence-related proteins p21^Cip1^, LaminB1, γH2AX, and HMGB1 in BV2 cells from D-gal+Ghrelin+oe-NC, D-gal+Ghrelin+oe-SHBG, and D-gal+Ghrelin+oe-SHBG+oe-Beclin1 groups. ***p<0.001.
